# Microglial transglutaminase 2 deficiency causes impaired synaptic remodelling and cognitive deficits in mice

**DOI:** 10.1111/cpr.13439

**Published:** 2023-03-06

**Authors:** Cong Liu, Xing Gao, Ruo‐Xi Shi, Ying‐Ying Wang, Xuan‐Cheng He, Hong‐Zhen Du, Baoyang Hu, Jianwei Jiao, Chang‐Mei Liu, Zhao‐Qian Teng

**Affiliations:** ^1^ State Key Laboratory of Stem Cell and Reproductive Biology Institute of Zoology, Chinese Academy of Sciences Beijing China; ^2^ Beijing Institute for Stem Cell and Regenerative Medicine Beijing China; ^3^ Institute for Stem Cell and Regeneration Chinese Academy of Sciences Beijing China; ^4^ Savaid Medical School University of Chinese Academy of Sciences Beijing China

## Abstract

Microglia are the primary source of transglutaminase 2 (TGM2) in the brain; however, the roles of microglial TGM2 in neural development and disease are still not well known. The aim of this study is to elucidate the role and mechanisms of microglial TGM2 in the brain. A mouse line with a specific knockout of *Tgm2* in microglia was generated. Immunohistochemistry, Western blot and qRT‐PCR assays were performed to evaluate the expression levels of TGM2, PSD‐95 and CD68. Confocal imaging, immunofluorescence staining and behavioural analyses were conducted to identify phenotypes of microglial TGM2 deficiency. Finally, RNA sequencing, qRT‐PCR and co‐culture of neurons and microglia were used to explore the potential mechanisms. Deletion of microglial *Tgm2* causes impaired synaptic pruning, reduced anxiety and increased cognitive deficits in mice. At the molecular level, the phagocytic genes, such as *Cq1a*, *C1qb* and *Tim4*, are significantly down‐regulated in TGM2‐deficient microglia. This study elucidates a novel role of microglial TGM2 in regulating synaptic remodelling and cognitive function, indicating that microglia *Tgm2* is essential for proper neural development.

## Graphical abstract

Microglial transglutaminase 2 (TGM2) is essential for regulating synaptic remodelling and cognitive function. Deletion of microglial *Tgm2* causes impaired synaptic pruning, reduced anxiety and increased cognitive deficits in mice. At the molecular level, the phagocytic genes, such as *Cq1a*, *C1qb* and *Tim4*, are significantly down‐regulated in TGM2‐deficient microglia.

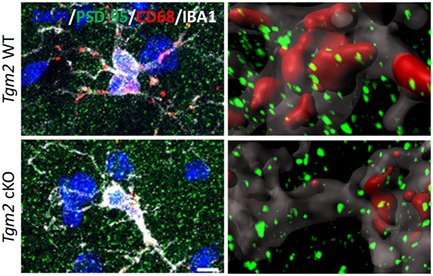

## 1 INTRODUCTION

Microglia are resident macrophages in the central nervous system (CNS) that play versatile roles in both health and disease.^1^ Originating from embryonic yolk sac, microglia migrate to the CNS during early embryonic development.^2^, ^3^ During the development, microglia interact with other cell types in the CNS to mediate developmental processes that are essential for achieving appropriate cellular architecture and function.^4^, ^5^ In adult CNS, microglia are highly ramified cells and are involved in the surveillance of the CNS parenchyma to maintain homeostasis.^6^ There is increasing evidence illustrating that microglia are also responsible for sculpting synaptic plasticity in the neural circuits of healthy brain.^7^, ^8^ Furthermore, microglia may in fact be initial factors for the onset of neurodegenerative and psychiatric diseases.^7^, ^9^, ^10^ Although the knowledge of microglial biology has grown exponentially in the past decade, we are still at the beginning stage to understand microglial roles in neurodegeneration and neurodevelopmental disorders.^11^, ^12^

Transglutaminases are a family of calcium‐activated enzymes that catalyse glutamine‐containing protein cross‐linking through a transamidation reaction.^13^, ^14^, ^15^ Of these enzymes, transglutaminase 2 (TGM2, also known as TG2) is the most abundant multifunctional transglutaminase in the CNS.^16^ TGM2 is localized both extracellularly and intracellularly and is also found in the nucleus in neuroblastoma cells.^17^ TGM2 has been shown to function as a deamidase, GTPase, protein disulphide isomerase or adapter/scaffold.^18^ In addition to enzymatic activity, TGM2 crosslinks proteins of extracellular matrix (ECM) by mediating the interaction of fibronectin with integrins.^19^, ^20^ Besides, TGM2 has been well established to act as a transcription factor to modulate gene expression.^21^

Emerging evidence suggests that TGM2 also plays pivotal roles in neural development and disease. Kjell et al. performed a comprehensive proteomic characterization of neurogenesis regions and identified that TGM2 is a crucial composition of neurogenic niche.^22^ TGM2 promotes differentiation and maturation of oligodendrocytes and is essential for myelination in CNS.^18^, ^23^, ^24^ Overexpression of TGM2 in cortical neurons results in reduced TrkB levels in the prefrontal cortex and a depressive‐like phenotype in mice.^16^, ^25^ In contrast, knockdown of TGM2 in primary neurons results in a significant loss of cell viability under oxygen–glucose deprivation (OGD) stress.^26^ However, knockdown of TGM2 in astrocytes significantly increases their viability following OGD.^27^ Therefore, TGM2 might play quite different roles in neurons and astrocytes.^16^ Interestingly, an RNA‐sequencing transcriptome of glia, neurons and vascular cells of the cerebral cortex clearly demonstrates that the mRNA level of *Tgm2* is much higher in microglia than that in neurons and other cells in mice.^28^ So far, the functions of microglial TGM2 in neural development and disease are still unknown.

In this study, to investigate the role of microglial TGM2 during neural development, we generated and analysed a mouse line with *Tgm2* knockout in microglia. We provide evidence that the deletion of microglial *Tgm2* causes impaired synaptic pruning, less anxiety and defects in cognition in mice.

## 2 METHODS

### 2.1 Mouse strains

All the animal procedures were approved by the Animal Committee of the Institute of Zoology Chinese Academy of Sciences, in accordance with national ethical guidelines and practice standards for animal care and use in research. *Cx3cr1‐Cre* mice (JAX:025524) were purchased from the Jackson Laboratory. Floxed *Tgm2* mice (*Tgm2* ^*fl/fl*^) were generated by Cyagen Biosciences (Suchow, China) using CRISPR/Cas9‐mediated genome editing (Figure S1). Two sgRNAs (matching forward strand of *Tgm2*: GACGTATAAACCTCACCGCAAGG; matching reverse strand of *Tgm2*: ATTCCCCGTGACTAGTGCGGGGG) were designed to separately target the distinct locations of the *Tgm2* introns 3 and 4. *Tgm2* ^*fl/fl*^ mice were crossed with the *Cx3cr1‐Cre* line to obtain conditional knockout mice of microglia *Tgm2* (*Tgm2* ^*fl/fl*^;*Cx3cr1‐Cre* ^+^, hereafter referred to as *Tgm2* cKO). Littermates with the genotype of *Tgm2* ^*fl/fl*^;*Cx3cr1‐Cre* ^−^ were used as controls (*Tgm2* wild type, hereafter referred to as *Tgm2* WT). Investigators were blinded to the genotype of mice for all experiments.

### 2.2 Immunohistochemistry and immunofluorescence

Immunohistochemistry (IHC) and immunofluorescence assays were performed as described previously.^29^ For immunostaining cultured cells, coverslips were fixed with 4% paraformaldehyde (PFA) for 20 min at room temperature. After blocking with 2% bovine serum albumin (BSA), coverslips were incubated with primary antibodies in blocking solution at 4°C overnight. The primary antibodies we used are as follows: TMEM119 (1:1000, Abcam, ab209064), IBA1 (1:1000, Novus, NB100‐1028) and MAP2 (1:1000, Biolegend, 822501). After washing with 1× phosphate buffer saline (PBS) three times for 10 min, coverslips were then incubated with secondary antibodies conjugated to Alexa Fluor 488 (1:1000, Invitrogen, A21206) and/or Alexa Fluor 647 (1:1000, Invitrogen, A21447) for 1 h at room temperature.

For IHC staining, 40‐μm thick brain sections were blocked in blocking solution (2% BSA, 0.3% Triton X‐100 and 0.2% sodium azide) for 1 h at room temperature and incubated overnight with the following primary antibodies: TGM2 (1:500, Abcam, Ab421), IBA1 (1:500, Wako, 019‐19741), PSD‐95 (1:500, ab2723, Abcam), NeuN (1:500 Millipore, ABN78) and/or CD68 (1:500, Bio‐rad, MCA1957GA). After washing with 1× PBS three times for 10 min, brain sections were incubated with secondary antibodies conjugated to Alexa Fluor 488 (1:1000, Invitrogen, A21206), Alexa Fluor 594 (1:1000, Invitrogen, A21209) and/or Alexa Fluor 647 (1:1000, Invitrogen, A21447). Finally, the sections were mounted with thin glass coverslips in the adhesion anti‐fade medium.

### 2.3 Image analysis

Images were obtained using a fluorescence microscope equipped with the digital camera (Zeiss LSM 880, Oberkochen, Germany). To measure PSD‐95 fluorescent intensity, Z‐stack images of PSD‐95 IHC‐stained sections containing both cortex and hippocampus from five pairs of 8‐week‐old Tgm2 WT and cKO littermates were captured at the same exposure time. Area, integrated density and mean grey values of at least 24 randomly selected frames (130 × 130 μm) in the cortex or hippocampi from 3 to 4 sections were measured for each animal. The mean fluorescence of small areas that had no fluorescence was calculated as the background reading for every image. The corrected total frame fluorescence was determined as the following equation: the corrected total frame fluorescence = integrated density − (area of selected frame × mean fluorescence of background readings). To quantify the numbers of microglia or neurons in the cortex and hippocampus, 24 randomly selected frames (650 × 650 μm) in the cortex or hippocampus from 4 sections were measured for each mouse. Five pairs of Tgm2 WT and cKO littermates at 8 weeks old were sampled. Cells were only counted when they were within a frame or on the right‐hand or bottom boundary line. Image analyses and quantification were performed using the ImageJ software (NIH). 3D morphometric measurements of microglia were recorded and analysed using the IMARIS software (Bitplane).

### 2.4 Behavioural assays

Mice were housed in groups of 2–4 per cage under cycles of 12‐h light/12‐h dark, with ad libitum access to water and food. For behavioural tests, we used the PASS software version 15.0.13 (NCSS, Kaysville) to estimate the appropriate animal sample sizes needed in each group. Male mice at 6–8 weeks old were moved to the testing room 24 h before test for acclimation. Experimental areas were cleaned with 70% ethanol before the behavioural testing and between subjects. All experiments were performed during the light phase. Videos were recorded and analysed using the software Smart V3.0.03 (Panlab, Barcelona, Spain).

#### 2.4.1 Nest building test

Nest building test was performed as described previously.^30^ Approximately 1 h before the dark phase, mice were transferred to individual testing cages containing 3.0 g cotton batting. Percentage of the nesting material used and the nesting score on a rating scale of 1–5 were assessed in the next morning. The nesting scores were determined as follows: score of 1, more than 90% of nestlet remaining intact; score of 2, about 50%–90% of nestlet remaining intact; score of 3, 50%–90% of nestlet has been shredded; score of 4, more than 90% of nestlet is torn, but the nest is flat; and score of 5, a (near) perfect nest with the shape of a crater.

#### 2.4.2 Open‐field test

Open‐field test (OFT) was performed as described previously.^31^ The OFT apparatus is a white plywood box (72 cm long × 72 cm wide × 36 cm tall). A central square (18 × 18 cm) was drawn in the middle of the open field chamber. Mice were randomly placed in a corner of the open field chamber and allowed to explore for 10 min. Total distance moved in the field and the number of centre entries were measured.

#### 2.4.3 Light/dark box test

Light/dark box test was conducted as described previously.^32^ Mice were placed in the dark compartment (18 × 27 cm) and facing the open door (7 × 7 cm) to the light compartment (27 × 27 cm) and allowed free exploration for 10 min. The time spent in the light compartment and the number of transitions between light and dark compartments were calculated.

#### 2.4.4 Barnes maze test

Barnes maze test (BMT) was performed in a 120 cm‐in‐diameter circular platform as described previously.^33^ Barnes maze has 20 evenly spaced holes (5 cm in diameter and 2 cm away from the edge) with only one hole leading to a removable hiding box located directly below the platform. The BMT procedure was composed of three phases: habituation (day 1), training (days 2 and 3) and probe (day 5). On day 1, mice were placed in the centre of the maze underneath a clear glass beaker while white noise was played. Mice were given 3 min to enter through the target hole into the hiding box after moving the glass beaker. Mice that did not find the hiding box by the end of 3 min were gently guided to the hiding box and allowed to stay in it for 30 s. In the training phase, mice were placed in an opaque box in the centre of the maze for 15 s. After moving the opaque box and turning on the buzzer, mice were allowed to explore the maze for 2 min. Three trials on training day 1 and two trials on training day 2 were conducted for every mouse. On the probe testing day, the hiding box was removed. Each mouse was given 2 min to search the escape hole during which the buzzer was turned on. Primary latency and number of visits to escape hole were recorded.

#### 2.4.5 Marble‐burying test

Mice were individually placed in the centre of propylene cages containing 5‐cm‐deep corncob granules, followed by overlaying 20 glass marbles (12 mm in diameter) equidistant in a 4 × 5 matrix. After a test session of 30 min, mice were removed from cages, and the number of marbles that were at least half‐covered with corncob granules was counted.

### 2.5 Western blotting

Total protein from brain tissues or from primary cultured microglia was extracted by RIPA buffer (Beyotime, P0013B). Samples were separated by SDS‐PAGE and immunoblotted using antibodies against TG2 (1:3000, Abcam) and PSD‐95 (1:3000; ab2723; Abcam). β‐Tubulin (1:5000, Easybio, BE3312‐100) or β‐actin (1:5000, Easybio, BE0033‐100) was used as a loading control.

### 2.6 Quantitative real‐time PCR

Total RNA was extracted with TRIzol (Invitrogen) and then reverse transcribed into cDNA using the TransScript One‐Step gDNA Removal and cDNA Synthesis SuperMix kit (TRAN, #20200725). Quantitative real‐time PCR (qPCR) was performed using the SYBR green PCR Master Mix (Yeasen, 11201ES08) and the Roche LightCycler@480II machine. Each reaction was run in triplicate, and glyceraldehyde‐3‐phosphate dehydrogenase (*Gapdh*) was used as an endogenous control for normalization. All primers used for qPCR are summarized in Table S1. The relative expression levels of genes were calculated by the ΔΔCT method.

### 2.7 Primary cell culture

#### 2.7.1 Neuronal culture

Mouse hippocampal neurons were isolated from pups at birth (P0) as described previously.^34^ Briefly, hippocampus was dissected and digested with TrypLE Express (Gibco, #12604013) for 10 min at 37°C. After terminating with the dulbecco's modified eagle medium (DMEM) containing 10% fetal bovine serum (FBS), single cell suspension was seeded into 24 well cell slides coated with poly‐d‐ornithine/laminin (PDL) and cultured in the neurobasal medium (Gibco, 21103049) containing 2% B‐27 supplemental (Gibco, 12587010), 1% penicillin–streptomycin solution (P/S, Hyclone, SV30010) and 1% GlutaMAX (Gibco, 35050061). Half of the culture medium was changed every 3 days.

#### 2.7.2 Microglial culture

Primary microglial cultures were performed according to our previous method.^35^ The cerebral cortex from P0 mice were dissected and digested with TrypLE Express. Trypsinization was stopped by adding the equal volume of the culture medium (DMEM/F12 medium containing 10% FBS and 1% P/S). Single cell suspension of glial cells was seeded in a sterile T‐75 flask containing 15 mL of the culture medium and cultured in a 5% CO_2_ incubator at 37°C for 8–10 days. The medium was replaced every 3 days. Non‐microglial cells were discarded by shaking the flask at 300 rps for 2 h and removing the supernatant. Highly purified microglia were resuspended in the culture medium and seeded on cell culture plates coated with PDL. For cell co‐culture experiments, *Tgm2* WT or cKO microglia were stained with DiI (Cell Plasma Membrane Staining Kit with DiI, Beyotime, C1991S) and then added to hippocampal neurons (16–17 days in vitro) at a microglia‐to‐neuron ratio of 1.5:1 for 24 h as previously described.^36^

### 2.8 Statistical analysis

Each of the experiments was evaluated normality with the Shapiro–Wilk for testing the null hypothesis that the sample values came from a normal distribution. Unpaired two‐tailed Student's *t*‐tests or ANOVA with post hoc tests were used to determine whether or not there was a statistically difference between the means of different groups using the GraphPad Prism 9.0 software. Differences at *p* < 0.05 were considered significant. All data are presented as mean ± SEM.

## 3 RESULTS

### 3.1 Absence of microglial *Tgm2* causes cognitive deficits in mice

By reanalysing a publicly available transcriptome database for purified neurons, microglia, astrocytes, oligodendrocyte precursor cells, newly formed oligodendrocytes, myelinating oligodendrocytes and endothelial cells from the P7 mouse cerebral cortex,^28^ we surprisingly found that microglia expressed a markedly higher level of *Tgm2* than any other cell types at P7 (Figure S1a). To validate this, we compared the expression levels of *Tgm2* in primary cultured microglia and neurons and did observe the highest mRNA level of *Tgm2* in microglia (Figure S1b). Consistently, co‐immunostaining of TGM2 and the microglia marker IBA1 in the P7 hippocampus demonstrated that the fluorescence signal intensity of TGM2 was stronger in IBA1^+^ microglial cells compared to nearby IBA1^−^ cells (Figure S1c). Given that *Tgm2* is highly expressed in microglia, we hypothesized that *Tgm2* may play a critical role in the microglial function in the brain.

To test the above hypothesis, we generated the microglial *Tgm2* knockout mice by intercrossing *Tgm2* ^*fl/fl*^ mice with the *Cx3cr1‐Cre* line (Figure S2a). qPCR analysis demonstrated that *Tgm2* mRNA was dramatically decreased in primary cultured microglia from *Tgm2* cKO mice (Figure S2b). As we expected, the TGM2 protein level was almost undetectable in primary cultured microglia from *Tgm2* cKO mice (Figure S2c,d), and the TGM2 protein level was significantly lower in hippocampi of *Tgm2* cKO mice compared with levels observed in WT littermates by the Western blotting analysis (Figure S2e,f). These data indicated that *Tgm2* was deleted in microglia in *Tgm2* cKO mice. To examine any possible phenotypes of *Cx3cr1* haploinsufficiency in microglia, we analysed the numbers of IBA1^+^ microglia, CD68^+^ structure and PSD‐95 puncta engulfed in the CD68^+^ structure by immunohistochemical staining of brain sections from *Cx3cr1‐Cre* ^+/−^ mice and their WT (*Cx3cr1‐Cre* ^−/−^) littermates at 8 weeks old. As shown in Figure S3, we did not observe appreciable effects of *Cx3cr1* haploinsufficiency on microglia number and synapse engulfment.

**FIGURE 1:**
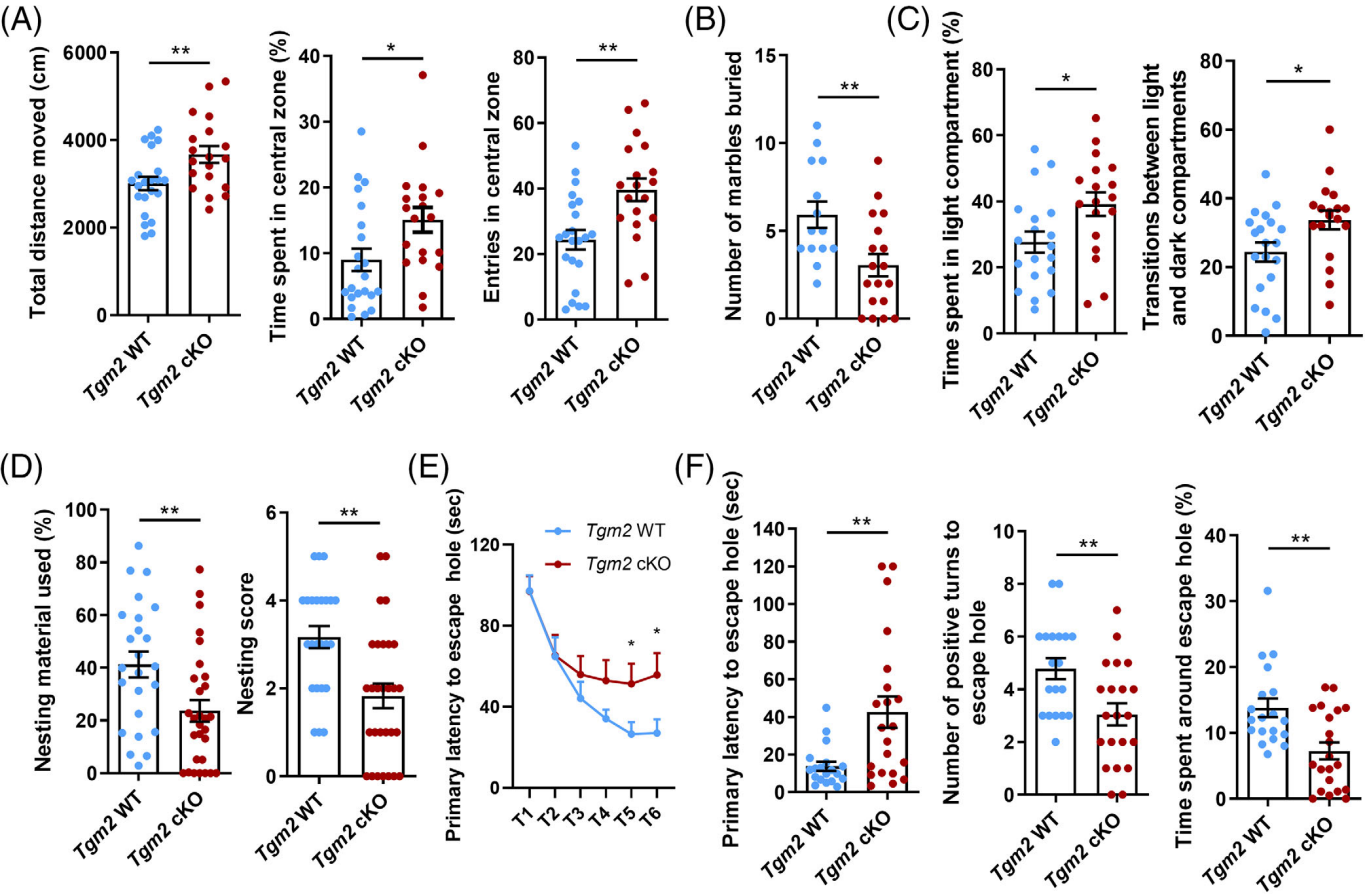
Microglial *Tgm2* deficiency causes reduced anxiety and increased cognitive deficits in mice. (A) Total distance moved, time spent in central zone and the number of entries in the central zone during the 10‐min session of the open field test. *n* = 19–22 male mice at 6–8 weeks old per group. (B) Number of marbles buried in the marble burying test. *n* = 14–18 male mice at 6–8 weeks old per group. (C) Time spent in light compartment and the number of transitions between light and dark compartments in light/dark box test. (D) Percentage of nesting material used and nesting score in the nesting building test. *n* = 24–29 male mice at 6–8 weeks old per group. (E) Primary latency to escape hole during the training phase of the Barnes maze test (BMT). (F) Primary latency and number of positive turns to escape hole and time spent around escape hole in the probe trial of the BMT. *n* = 19–21 male mice at 6–8 weeks old per group. Data are presented as mean ± SEM. *p < 0.05; **p < 0.01.

To investigate the function of microglial *Tgm2* in the brain, we first conducted a series of behavioural tests, including OFT, light/dark box test, BMT, nest building test and marble‐burying test, to examine whether *Tgm2* cKO male mice display any abnormal behavioural features. In the OFT, total distance moved, an index of activity, was significantly increased in *Tgm2* cKO male mice compared with that in WT male mice (Figure 1A). Meanwhile, *Tgm2* cKO male mice had more entries and spent more time in the centre zone (Figure 1A). These data suggested that *Tgm2* cKO male mice exhibited a phenotype of hyperactivity and less anxiety. Next, we conducted marble‐burying test and light/dark box test to further assess anxiety. There were fewer marbles buried in *Tgm2* cKO male mice in the marble burying test, while a longer time spent in the light compartment and a larger number of transitions between light and dark compartments were detected in *Tgm2* cKO male mice in the light/dark box test (Figure 1B,C). These findings confirmed that *Tgm2* cKO male mice had less anxiety.

Since anxiety‐like behaviour is closely related to cognition,^37^ we next investigated whether cognitive function is affected in *Tgm2* cKO male mice. In nest building test, *Tgm2* cKO male mice tore a smaller part of nestlets and constructed nests of poorer quality as compared to WT male mice (Figure 1D). In BMT, we observed that *Tgm2* cKO male mice displayed a significant delay to locate the hiding box in both training and probe phases (Figure 1E,F), and they also exhibited fewer positive turns to escape hole and spent less time wandering around the escape hole (Figure 1F). Altogether, these behavioural data strongly support that microglial *Tgm2* is essential for the cognitive function.

### 3.2 Loss of microglial *Tgm2* leads to increased expression of postsynaptic density protein PSD‐95

**FIGURE 2:**
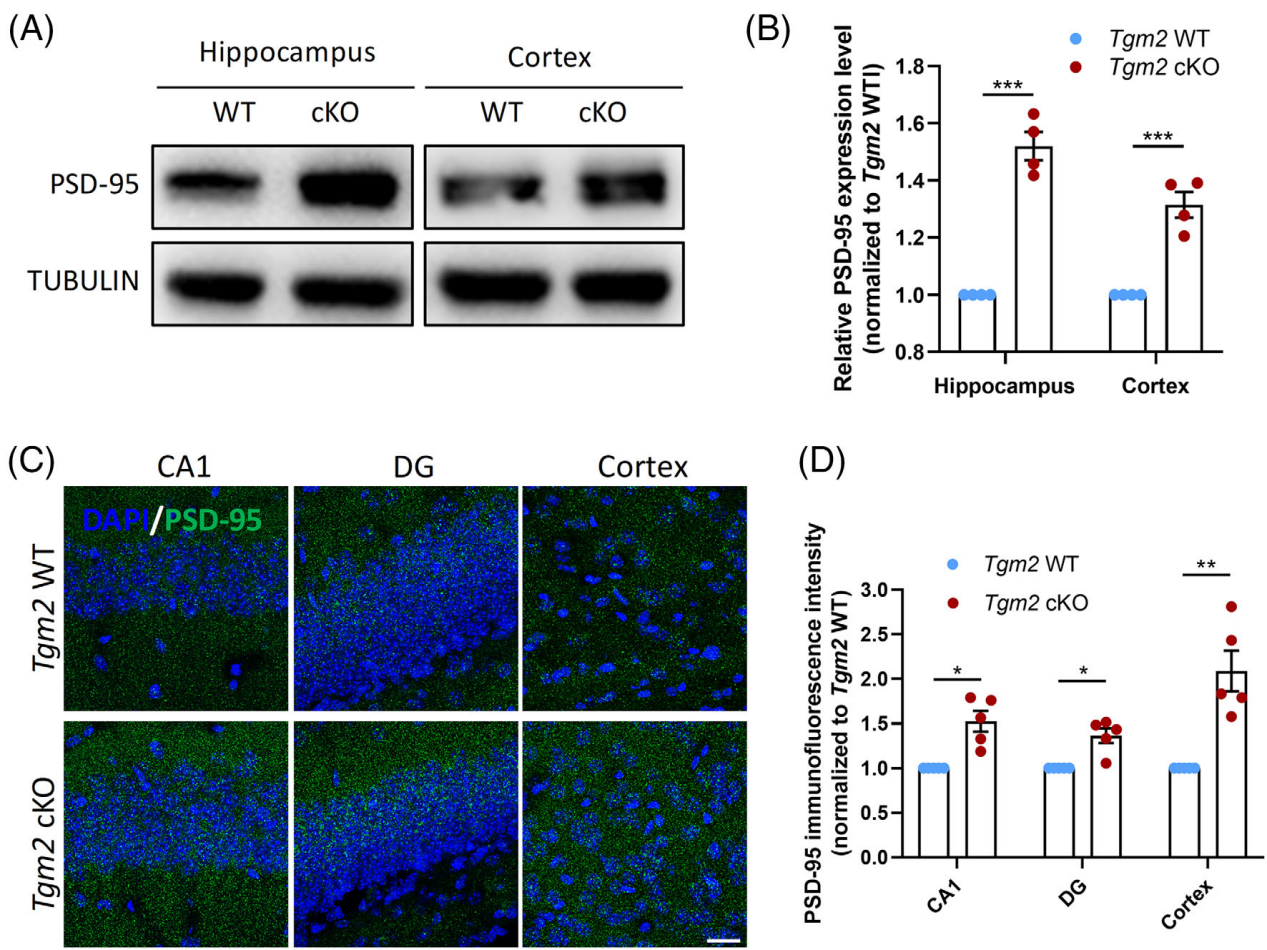
Loss of microglial *Tgm2* leads to increased expression of PSD‐95. (A) Representative images of Western blot for PSD‐95 in hippocampal and cortical tissues of *Tgm2* cKO and WT male mice at 8 weeks old. (B) Quantification of PSD‐95 protein expressions in hippocampal and cortical tissues. (C) Representative images of immunohistochemical staining of PSD‐95 in hippocampus and cortex. CA1, cornu ammonis 1; DG, dentate gyrus. Scale bar, 20 μm. (D) Quantification of relative immunofluorescence intensity of PSD‐95 in both hippocampus and cortex. Data are presented as mean ± SEM; *n* = 4–5 animals. *p < 0.05; **p < 0.01; ***p < 0.001.

Considering that synapse remodelling is mediated by microglia,^1^ to explore the potential mechanisms of cognitive deficit in microglial *Tgm2* knockout mice, we examined the expression levels of PSD‐95 in hippocampal and cortical tissues of both 8‐weeks‐old *Tgm2* cKO and WT mice by Western blot and IHC assays. Western blot analysis revealed a significant upregulation of PSD‐95 protein levels in both hippocampus and cortex of *Tgm2* cKO mice (Figure 2A,B). Consistently, increased fluorescence intensities of PSD‐95 in the cortex, cornu ammonis 1 (CA1) and dentate gyrus (DG) regions were verified by IHC analysis (Figure 2C,D). Taken together, these results suggest that microglial *Tgm2* may play a key role in regulating synapse remodelling.

### 3.3 Microglial TGM2 is required for synapse engulfment in vivo

There was no difference in the number of IBA1^+^ microglia in the cortex and hippocampus between 8‐week‐old *Tgm2* cKO and WT mice (Figure S4a,b). Quantification of the total length of all process, process volume and branch number did not find any morphological difference between 8‐week‐old *Tgm2* cKO and WT microglia by IMARIS‐based 3D morphometric measurements (Figure S4c,d). Moreover, immunohistochemical staining of a neuronal marker NeuN showed that the neuron densities in the hippocampus and cortex of *Tgm2* cKO were the same as WT mice (Figure S5a,b). These results indicate that *Tgm2* loss‐of‐function does not alter the density and morphology of microglia as well as the number of neurons in vivo.

**FIGURE 3:**
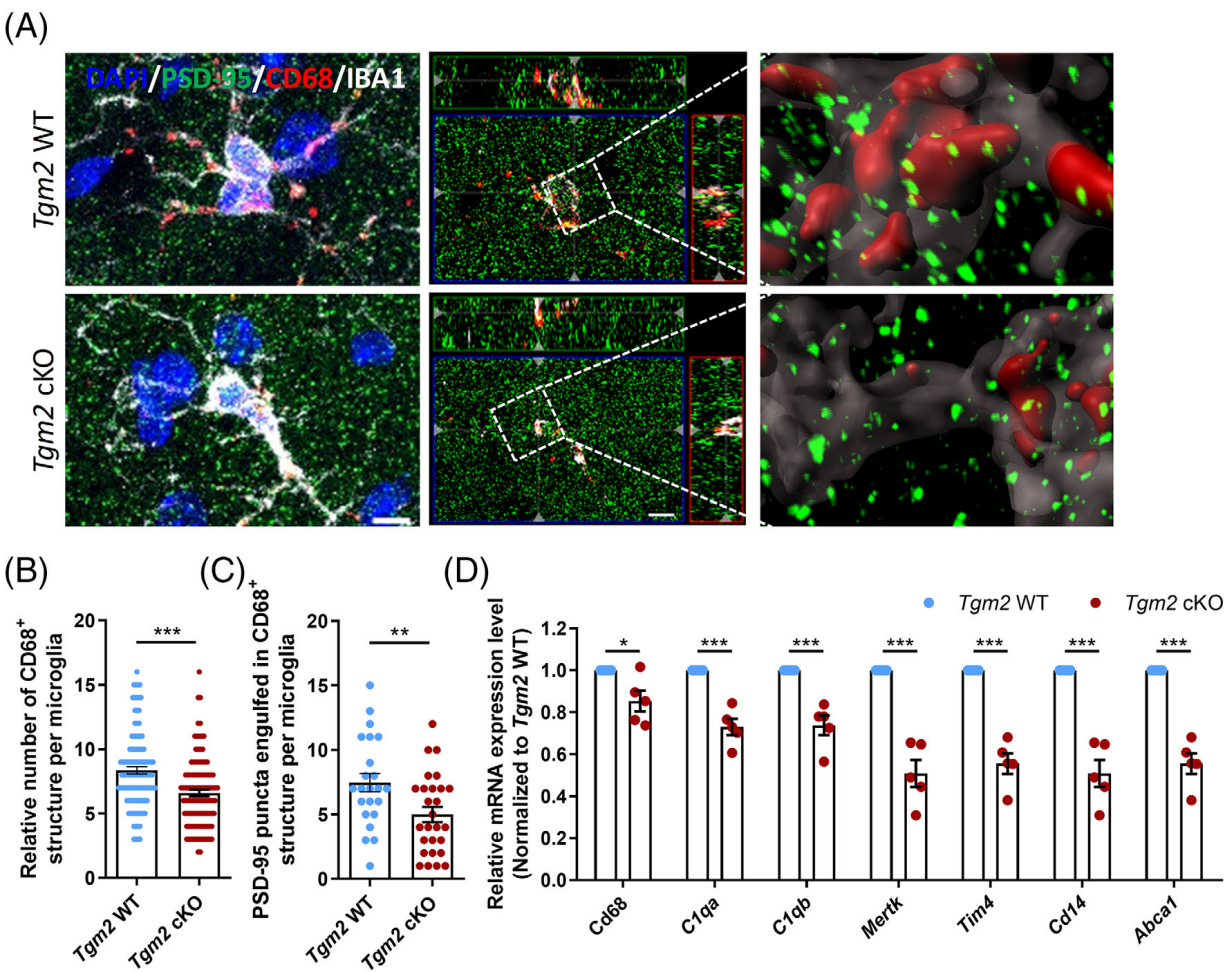
Microglial *Tgm2* is required for synapse engulfment in vivo. (A) Representative 3D surface rendering images showing volume reconstruction of IBA1^+^ microglia,^10^ CD68^+^ structure (red) and PSD‐95^+^ puncta (green). Scale bar, 5 μm. (B) Quantification of the number of CD68^+^ structures per microglia. *n* = 107–110 microglia from five male mice at 8 weeks old per group. (C) Quantification of the number of PSD‐95^+^ puncta engulfed within the CD68^+^ structure per microglia. *n* = 23–27 microglia from five male mice at 8 weeks old per group. (D) Relative mRNA expression levels of genes related to phagocytosis in hippocampal tissue by the quantitative real‐time PCR analysis. *n* = 5 animals. Data are presented as mean ± SEM. *p < 0.05; **p < 0.01; ***p < 0.001.

However, IHC staining for PSD‐95, CD68 and IBA1 revealed a reduced number of CD68^+^ structures (Figure 3A,B) and PSD‐95 puncta engulfed in CD68^+^ structures (Figure 3C) per hippocampal microglia in *Tgm2* cKO mice compared with WT littermates at 8 weeks old. Next, we performed qPCR analysis and discovered that the expression of phagocytic markers (CD68 and CD14) and phagocytic genes, such as *Cq1a*, *C1qb*, *Mertk*, *Tim4* and *Abca1*, were significantly decreased in hippocampal tissues in *Tgm2* cKO mice (Figure 3D). These data support that the deletion of *TGM2* in microglia inhibits synapse engulfment in vivo.

### 3.4 Deficiency of microglial TGM2 reduces synapse phagocytosis in vitro

**FIGURE 4:**
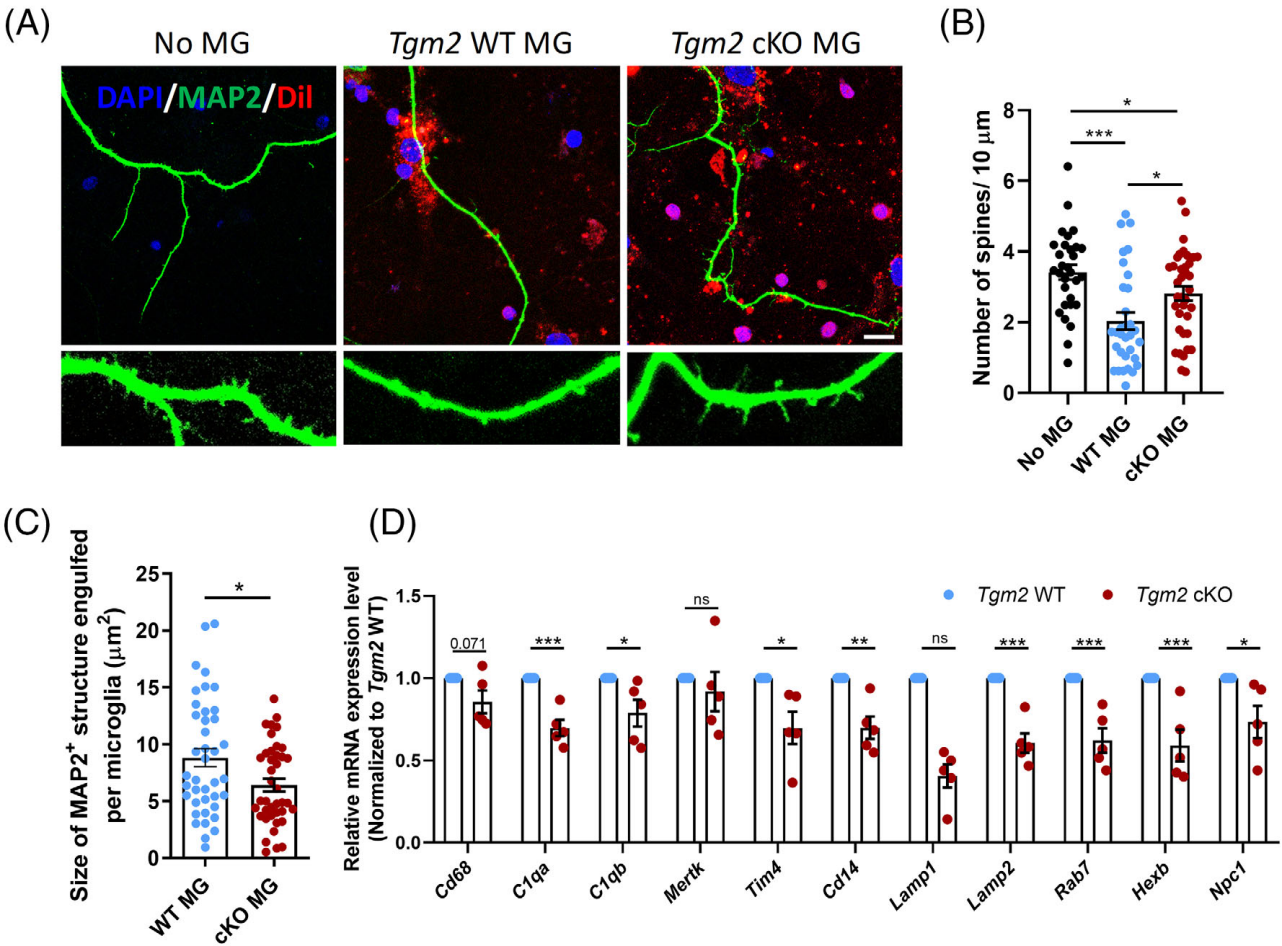
Deficiency of microglial TGM2 reduces synapse phagocytosis in vitro. (A) Representative images of dendritic spines of primary hippocampal neurons (MAP2^+^, green) cultured alone (no microglia [MG]) or co‐cultured with either WT or *Tgm2* cKO microglia (Dil^+^, red) for 24 h. Scale bar, 10 μm. (B) Quantification of spine numbers of primary cultured hippocampal neurons. *n* = 30–37 neurons from three males. (C) Quantification of relative size of MAP2^+^ structure engulfed per Dil‐labelled microglia. *n* = 40 microglia from three males. (D) Relative mRNA expression levels of genes related to phagocytosis and endocytosis in microglia by quantitative real‐time PCR analysis. *n* = 5 cultures. Data are presented as mean ± SEM. *p < 0.05; **p < 0.01; ***p < 0.001.

The fluorescent probe Dil, a cell plasma membrane dye, is commonly used as a safe, inexpensive and fast method to label and trace cells.^38^ We found that all live cells could be stained with Dil and almost all of Dil^+^ cell (>96%) were TREM119^+^IBA1^+^ microglia, indicating the high purity of our primary cultured microglia (Figure S6a,b). To validate the reduced phagocytosis feature of *Tgm2* cKO microglia, we stained primary microglia with Dil and then added them into hippocampal neuronal cultures (16–17 days in vitro) at a microglia‐to‐neuron ratio of 1.5:1 for 24 h as previously described.^31^, ^36^ We observed that there was a significant reduction in spine numbers of hippocampal neurons in contact with *Tgm2* cKO microglia (Figure 4A,B). Meanwhile, the relative size of the MAP2^+^ structure engulfed per *Tgm2* cKO microglia was significantly smaller than that of *Tgm2* WT microglia (Figure 4C). On the molecular level, the phagocytic genes, such as *Cq1a*, *C1qb*, *Tim4*, *Lamp1*, *Lamp2*, *Rab7*, *Hexb1* and *Npc1*, were down‐regulated in *Tgm2* cKO microglia compared to WT controls (Figure 4D). Again, these in vitro data strongly suggest that microglia TGM2 is required for proper synapse remodelling.

## 4 DISCUSSION

Learning and memory require the formation of new neural circuits in the brain.^39^ Microglia play critical roles in learning and memory as they regulate the formation and stability of synapses and modulate synaptic plasticity by releasing brain derived neurotrophic facor (BDNF) or inflammatory molecules to communicate with neurons.^40^ This study elucidates a novel role of microglial TGM2 in regulating synaptic remodelling and cognition under physiological conditions. The absence of microglial TGM2 causes reduced anxiety and increased cognitive deficits in mice. It is worth noting that microglia are the primary source of TGM2 in mice during oligodendrocyte development, and microglial TGM2 promotes myelination and myelin repair via GPR56/ADGRG1 in a focal de‐/re‐myelination model using injections of lysophosphatidylcholine into the external capsule of the mouse corpus callosum.^23^ Since oligodendrocytes and myelin play key roles during and/or following learning,^41^ we cannot exclude the possibility that impaired myelination might also contribute to cognitive dysfunction we observed in mice with microglial *Tgm2‐*deletion, but exactly how abnormal myelination influences the underlying neural circuits remains to be investigated in the future.

TGM2 is an enzyme that has multifunctional activity of crosslinking, deamidation, GTPase and ATPase, isopeptidase, as well as adapter/scaffold activity.^42^ TGM2 accumulation causes cell death in neuroblastoma cells,^43^ hepatocytes^44^ and neurons.^45^ However, a prosurvival role of TGM2 has also been reported in injured liver^46^ as well as in brain with ischaemia and stroke.^47^, ^48^ Indeed, the function that TG2 takes on relies upon its different domains and conformations, cell types, intracellular localization and extracellular environment.^16^ Although our study supports that the deficiency of microglial TGM2 does not affect the numbers of microglia and neurons in the hippocampus and cortex under normal conditions, it will be very interesting to examine whether there is a difference in cell death and survival in the brain following acute injury or stress.

Synapse formation, stabilization and elimination are indispensable mechanisms underlying synaptic plasticity, which is associated and mediates learning and memory.^49^ Microglia mediate synaptic pruning and cognition, but only a few molecular mechanisms have been identified, including CX3CR1,^50^ TREM2,^36^ EZH2,^51^ EED^31^, ^52^ and the complement cascade (C1q, C3 and CR3).^53^ A previous study has shown that the clearance of apoptotic cells is defective in *Tgm2* ^−/−^ mice, because the lack of TGM2 prevents the production of active transforming growth factor‐β1 in macrophages exposed to apoptotic cells, which is required for efficient phagocytosis of apoptotic bodies.^54^ Consistently, our study unveils an important role for microglial TGM2 in the control of engulfment of synapses. Although, at present, the molecular mechanism of how the deletion of microglial *Tgm2* leads to reduced anxiety and increased cognitive deficits still remains elusive, we have identified the phagocytic genes, such as *Cq1a*, *C1qb* and *Tim4*, to be down‐regulated in *Tgm2* cKO microglia compared with WT controls. These results help explain the key regulatory role of microglial TGM2 in synaptic remodelling.

Synapse and synaptic protein loss are fundamental to the pathophysiology of Alzheimer's disease (AD), frontotemporal dementia, ischaemic vascular dementia and spongiform encephalopathy.^55^ Moreover, in the human AD brain, there is strong evidence for activation of complement^56^ and for microglia‐mediated synapse loss via the complement pathway in AD.^57^ Since our data indicate that TGM2 deficiency causes reduced synapse phagocytosis, enhanced expression of postsynaptic density protein PSD‐95 and down‐regulated expression of complement (*Cq1a* and *C1qb*), it will be interesting to investigate whether blocking the microglial Tgm2 pathway has a therapeutic potential for synapse damage and loss in the context of neurodegenerative diseases.

It has been recently recognized that microglia possess sexually dimorphic roles in the developing, adult and aged brain that have been associated with neurological disorders such as autism and traumatic brain injuries.^35^, ^58^, ^59^, ^60^, ^61^ The adult female mice, under physiological conditions, are usually exposed to short‐term changes in gonadal hormones levels (estrous cycle). A recent study demonstrates that neuronal chromatin organization in the female ventral hippocampus of mouse fluctuates with the estrous cycle and that chromatin organizational changes associate with the transcriptional activity of genes important for neuronal function and behaviour.^62^ To avoid possible effects of the phase of female estrous cycle on behaviours,^63^ we only used male mice in the present study. It would be interesting to know whether *Tgm2* cKO‐induced changes in synaptic remodelling and cognitive function are sex‐specific at different developmental stages and/or under various disease conditions using various methods like behavioural, electrophysiological, immunohistological and molecular assays.

It is worthy to note that *Cx3cr1‐Cre* can sometimes evince haploinsufficiency in mouse models under pathological conditions, including LPS‐ or acute injury‐induced systemic inflammation, MPTP‐induced parkinsonism and the *SOD1* ^G93A^ model of amyotrophic lateral sclerosis.^64^, ^65^, ^66^ In a previous study, Paolicelli and colleagues observed a transient deficit in synaptic pruning and a transient increase in dendritic spine density in the *Cx3cr1* knockout (KO/KO) mice, not in *Cx3cr1* ^*GFP/+*^ reporter mice (KO/+), suggesting that *Cx3cr1* haploinsufficiency may not have obvious effects on synaptic pruning by microglia during normal brain development.^67^ In this study, we also do not detect any obvious effects of *Cx3cr1* haploinsufficiency on the microglia number and synapse engulfment. To ascertain a direct effect of reduced synapse phagocytosis on TGM2‐knockout microglia, additional experiments are needed, that is, blocking phagocytosis of microglia under WT conditions, rescuing TGM2‐knockout phenotype and comparing to other cell‐type specific knockouts. In addition, CX3CR1 is a chemokine receptor expressed in microglia, as well as the mononuclear phagocyte system, it is unclear whether *Tgm2* knockout in macrophages, monocytes or neutrophils will contribute to the pathogenesis of neurological diseases.

In conclusion, our findings suggest that microglial TGM2 is essential for proper synaptic remodelling and cognitive function. Given the complex heterogeneity of microglia phenotypes and the multifunctional activity of TGM2 across different types of cells, future investigations are required to elucidate the exact localization of TGM2 in various microglial subtypes as well as the molecular mechanisms underlying the functional phenotypes of TGM2 in health and disease.

## AUTHOR CONTRIBUTIONS

Zhao‐Qian Teng and Cong Liu conceived and designed the research. Cong Liu and Zhao‐Qian Teng drafted the manuscript. Cong Liu, Xing Gao, Ruo‐Xi Shi, Ying‐Ying Wang, Xuan‐Cheng He and Hong‐Zhen Du performed the experiments and approved the final version of the manuscript. Baoyang Hu, Jianwei Jiao, Zhao‐Qian Teng and Chang‐Mei Liu supervised the research and revised the manuscript.

## CONFLICT OF INTEREST STATEMENT

The authors declare no competing interests.

## Supporting information


**Data S1:** Supporting Information

## Data Availability

All data generated and analyzed are included in this article. Further enquiries can be directed to the corresponding author.
