# Supplementary material for: Microglial transglutaminase 2 deficiency causes impaired synaptic remodelling and cognitive deficits in mice
Source: Cell Prolif. 2023 Mar 6;56(9):e13439. doi: 10.1111/cpr.13439 (PMC10472527; doi:10.1111/cpr.13439)
Supplement: Supplementary file 1 — Data S1: Supporting Information [file CPR-56-e13439-s001.docx]

# **Supplemental information**

## **Supplemental figures**


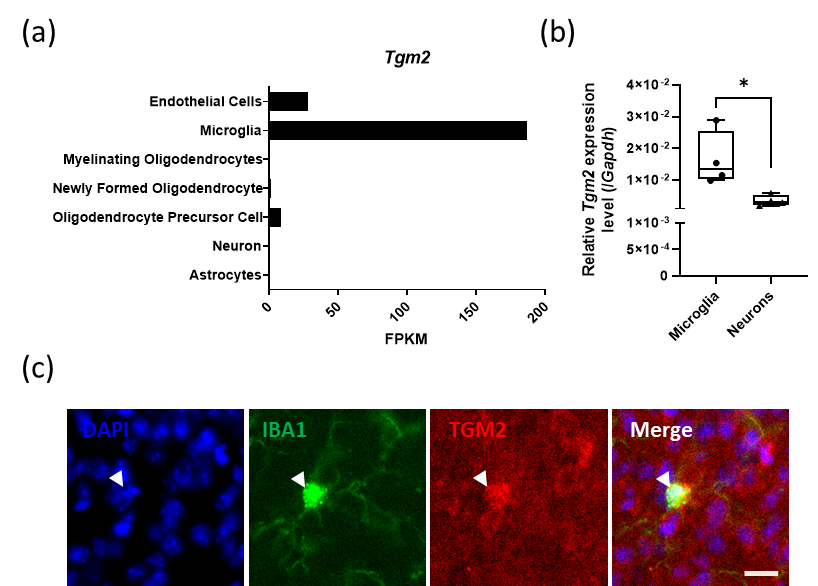


### **Figure S1. Tgm2 is highly expressed in microglia.**

(a) Data mining of publicly available databases (https://[www.brainrnaseq.org/](http://www.brainrnaseq.org/)) indicated that *Tgm2* was highly expressed in purified microglia at P7 (Zhang et al., 2014). (b) Quantitative real-time PCR analysis showed that the mRNA expression level of *Tgm2* was higher in primary cultured microglia than that in cultured neurons. *n* = 4. Data are presented as mean ± SEM. **P* < 0.05. (c) Co-immunostaining of TGM2 (red) and the microglia marker IBA1 (green) in P7 hippocampus. Arrowheads indicate the colocalization of TGM2 and IBA1. Scale bar, 20 μm.


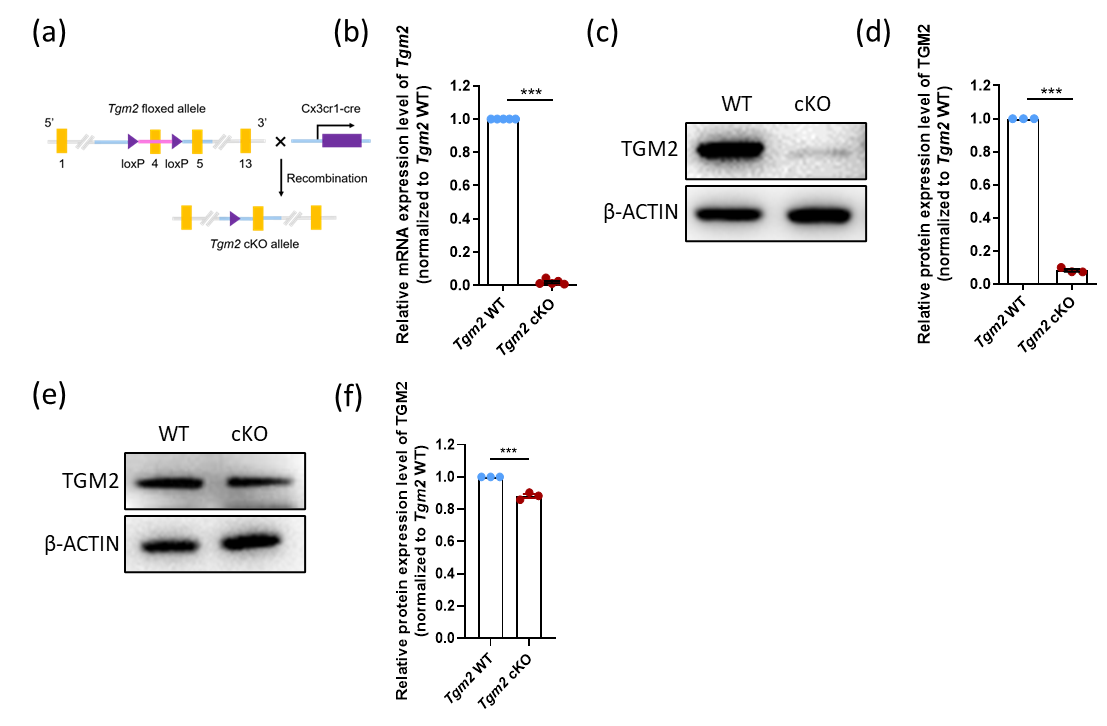


### **Figure S2. Generation and validation of Tgm2 cKO mice.**

(a) Schematic diagram for the generation of *Tgm2* cKO mice. Exon 4 of the *Tgm2* allele is flanked by LoxP sites, allowing for *Cre* recombinase-mediated deletion of exon 4. (b) Quantitative real-time PCR analysis revealed that *Tgm2* mRNA was significantly reduced in primary cultured microglia from *Tgm2* cKO mice. *n* = 5 cultures from different mice. (c) Representative images and (d) quantification of Western blot analysis indicated that TGM2 protein level was significantly decreased in primary cultured microglia from *Tgm2* cKO mice. *n* = 3 cultures from different mice. (e) Representative images and (f) quantification of Western blot analysis demonstrated that TGM2 protein level was significantly lower in hippocampi of *Tgm2* cKO mice compared with levels observed in WT mice. *n* = 3 mice per group. Data are presented as mean ± SEM. ****P* < 0.001.


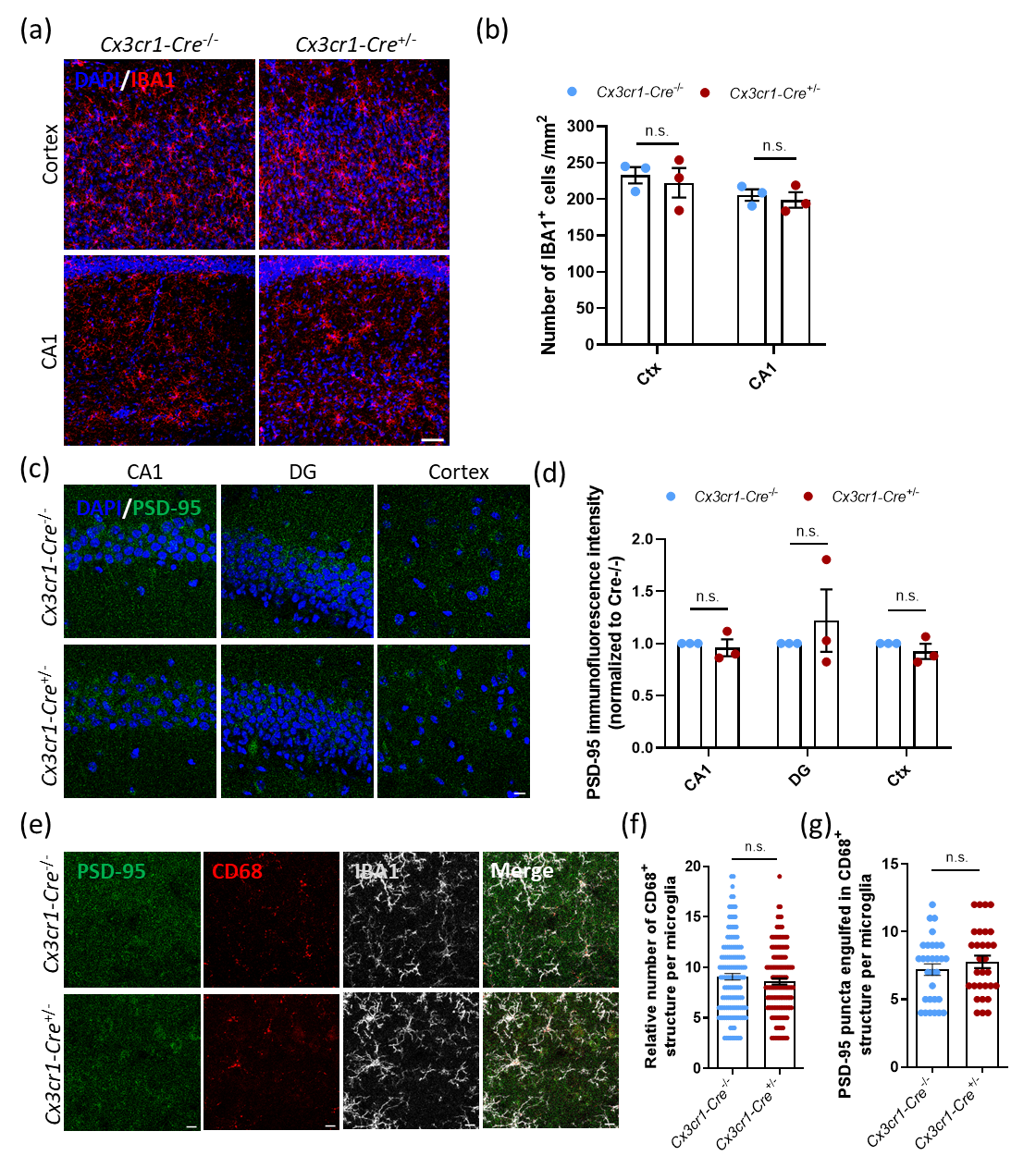


### **Figure S3. Cx3cr1 haploinsufficiency has no appreciable effects on microglia number and synapse engulfment.**

(a) Representative images and (b) quantification of IBA1 immunostaining demonstrated that *Cx3cr1-Cre*^+/-^ mice had the same number of IBA1^+^ microglia as that of their WT (*Cx3cr1-Cre*^-/-^) littermates at 8 weeks old. Scale bar, 50μm. (c) Representative images and (d) quantification of PSD-95 immunostaining showed that there was no difference in PSD-95 immunofluorescence intensity between *Cx3cr1-Cre*^+/-^ mice and their WT littermates at 8 weeks old. Scale bar, 20μm. (e, f, g) Representative images (e) and quantifications of PSD-95, CD68 and IBA1 immunostainings showed that *Cx3cr1* haploinsufficiency has no obvious effects on the numbers of CD68^+^ structure (f) and PSD-95 puncta engulfed in CD68^+^ structure (g). Scale bar, 10μm. *n* = 3 males per group. Data are presented as mean ± SEM. n.s., non-significant.


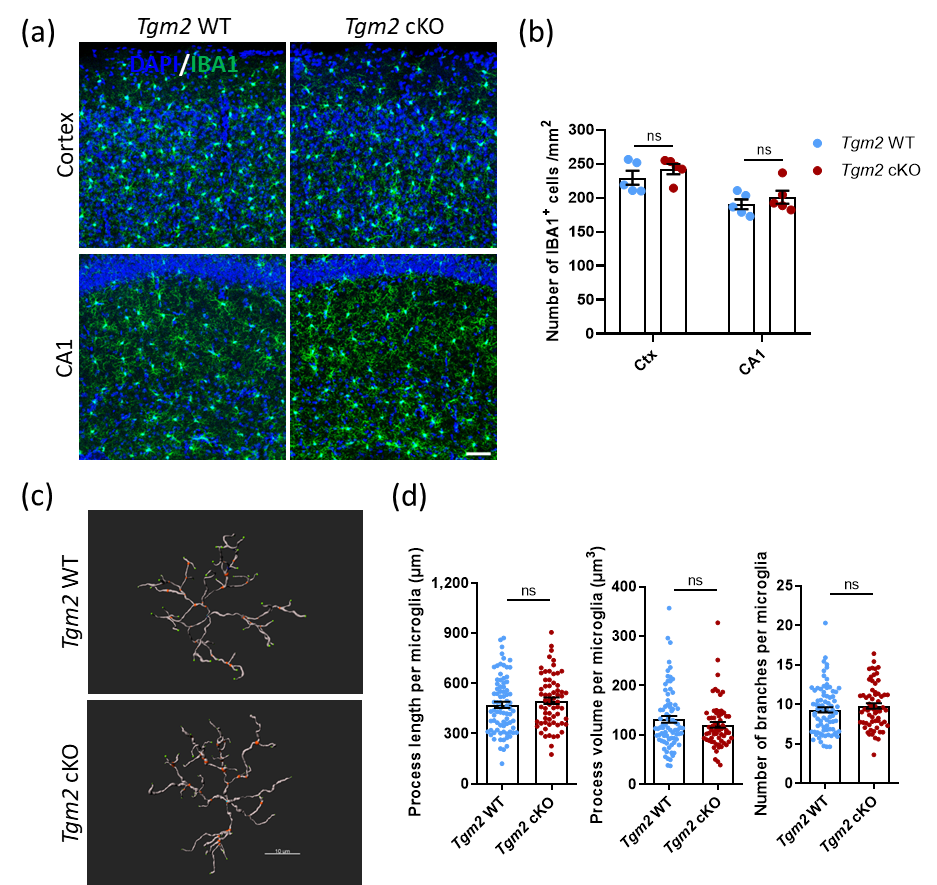


### **Figure S4. Genetic ablation of *Tgm2* does not affect the number and morphology of microglia.**

(a) Representative images of Iba1 immunohistochemical staining in hippocampus and cortex. Scale bar, 50μm. (b) Quantification of microglia (IBA1^+^, green) in hippocampus and cortex. *n* = 5 mice per group. (c) Representative images of 3D reconstruction of hippocampal microglia. Scale bar, 10μm. (d) Quantification of process length, process volume and branch number of microglia by IMARIS-based 3D morphometric measurements. *n* = 64-80 microglia from 5 mice. Data are presented as mean ± SEM. ns, non-significant.


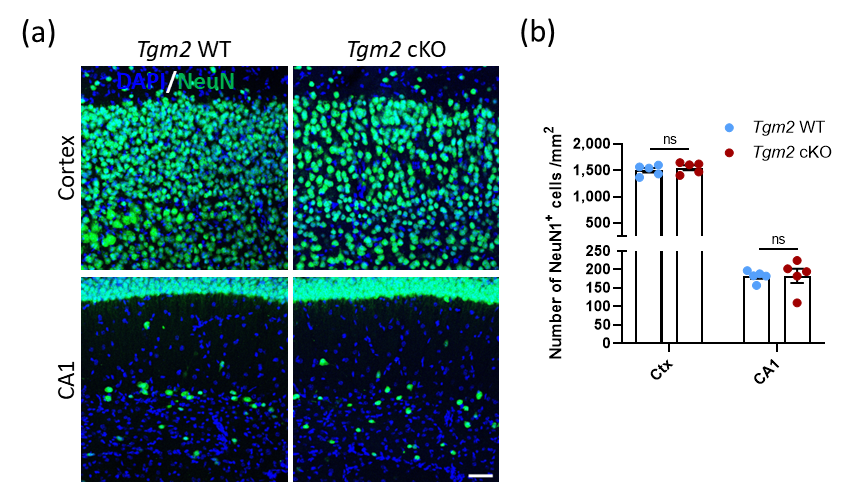


### **Figure S5. Microglia-specific deletion of *Tgm2* does not affect the number of neurons.**

(a) Representative images of NeuN immunohistochemical staining in hippocampus and cortex. Scale bar, 50μm. (b) Quantification of neurons (NeuN^+^, green) in hippocampus and cortex. *n* = 5 mice per group. Data are presented as mean ± SEM. ns, non-significant.


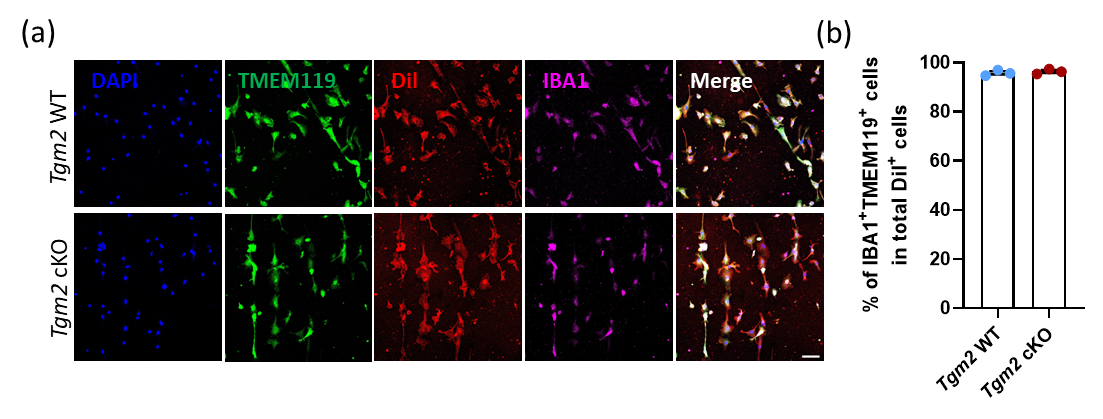


### **Figure S6. Validation of microglial purity of Dil-labeled cells.**

(a) Representative images of co-staining of Dil (red), IBA1 (purple) and TMEM119 (green). Scale bar, 50μm. (b) Quantification of the percentage of IBA1^+^TMEM119^+^ microglia among total Dil^+^ cells.

## **Supplemental Table 1**

All primers used for qPCR
